# An Optimized Trajectory Planner and Motion Controller Framework for Autonomous Driving in Unstructured Environments

**DOI:** 10.3390/s21134409

**Published:** 2021-06-27

**Authors:** Lu Xiong, Zhiqiang Fu, Dequan Zeng, Bo Leng

**Affiliations:** 1 School of Automotive Studies, Tongji University, Shanghai 201804, China; xiong_lu@tongji.edu.cn (L.X.); 1811480@tongji.edu.cn (Z.F.); 1610849@tongji.edu.cn (D.Z.); 2 Clean Energy Automotive Engineering Centre, Tongji University, Shanghai 201804, China

**Keywords:** autonomous driving, trajectory planner, obstacle avoidance, motion controller, model predictive control

## Abstract

This paper proposes an optimized trajectory planner and motion planner framework, which aim to deal with obstacle avoidance along a reference road for autonomous driving in unstructured environments. The trajectory planning problem is decomposed into lateral and longitudinal planning sub-tasks along the reference road. First, a vehicle kinematic model with road coordinates is established to describe the lateral movement of the vehicle. Then, nonlinear optimization based on a vehicle kinematic model in the space domain is employed to smooth the reference road. Second, a multilayered search algorithm is applied in the lateral-space domain to deal with obstacles and find a suitable path boundary. Then, the optimized path planner calculates the optimal path by considering the distance to the reference road and the curvature constraints. Furthermore, the optimized speed planner takes into account the speed boundary in the space domain and the constraints on vehicle acceleration. The optimal speed profile is obtained by using a numerical optimization method. Furthermore, a motion controller based on a kinematic error model is proposed to follow the desired trajectory. Finally, the experimental results show the effectiveness of the proposed trajectory planner and motion controller framework in handling typical scenarios and avoiding obstacles safely and smoothly on the reference road and in unstructured environments.

## 1. Introduction

The popularity of autonomous technology is bringing profound changes to all walks of life. This intelligent technology is also expanding the scope of mobile robot operations from indoors to outdoors, from closed areas to semi-closed and, even, fully open areas [1]. More and more practical technologies are being applied in low-speed vehicles, such as autonomous logistics vehicles and autonomous sweepers. These vehicles are usually driven on unstructured roads and complete some specific tasks. In order to achieve excellent completion of the job requirements and be collision-free, the trajectory planner plays an important role in generating a real-time feasible trajectory, while motion controllers have the core responsibility for taking robust and stable action [2].

Many advanced path planning technologies have been applied for autonomous agents in unstructured environments, such as the graph search method [3], random sampling method [4], optimal method [5,6], and artificial potential field [7,8]. Among them, the search-based method is the most widely used method in the field of mobile robots, such as Dijkstra and A star algorithms, which aim to solve the shortest path problem [9]. Dmitri Dolgov [10,11] associated the continuous state with each cell by considering the minimum turning radius, and then improved the quality of the planned path solution through nonlinear numerical optimization; the planning results are better applied to unstructured roads. Ricardo [12], based on a graph search, created a kinematically feasible high-resolution lattice state space for non-holonomic robots. It reduced planning time by using a set of offline pre-calculated maps. However, the planning frequency was lower and it failed to consider the continuity of curvature directly. Among the random sampling-based algorithms, the rapidly-exploring random tree (RRT) is widely used in unstructured environments. It is constructed incrementally from a randomly selected point to the expected distance of the tree [13]. Although it has probabilistic completeness, it is difficult to find the optimal solution by searching unstructured roads. The artificial potential field method is often used for unstructured roads due to its low computation and strong ability for multiple information fusion [14,15]. The basic principle of the artificial potential field is that the host agent travels in the direction with the least net force under attraction of the objects (such as target node, reference path and so on) and the repulsion of the obstacles (such as barrier, road curb and so on) [16,17]. According to the attraction potential field, to make the robot approach the target, the repulsive potential field makes the robot stay away from obstacles, the two potential fields are superimposed to form a virtual potential field of robot motion, and then the shortest path to the target position is searched based on the gradient change of the potential field. Wang et al. [18] combined a virtual-leader potential field and a vehicle–vehicle potential field to generate a path for UGVs formation, and the potential field function was set as an ellipse. The artificial potential field method can assign different potential functions to different types of obstacles and road structures, and plan paths based on these potential functions. However, this algorithm can be trapped in local minima during the process of path planning.

Compared to trajectory planning on a structured road, the main challenges of autonomous driving in unstructured environments are that the obstacle shapes are irregular, and lane boundary constraints do not exist. There are some literature reports solving trajectory planning problems in unstructured environments. Li et al. [19] proposed a rollover-free local path planning algorithm for an off-road autopilot in different road conditions, which used a 3D grid map and generated a series of 3D path candidates for the road coordinates. The optimal path is selected considering the rollover prevention and the cost of safety. Chu K [20] applied a sampling-based trajectory planner by using the cubic spline to avoid static obstacles. The sampling-based trajectory planner aims to select a best trajectory from a rich set of suboptimal candidates. Li X [21] used a model-based predictive trajectory planner by using cubic spirals. The multilayer terminal states are sampled to generate more candidate paths for autonomous ground vehicles. These path candidates are evaluated through the designed objective function, which considers the smoothness, deviation from the reference path, and consistency of the path. At the same time, the velocity planning is calculated using a trapezoidal curve considering the acceleration of the vehicle. Zhang Y [22] proposed a multi-phase deterministic state-space sampling method to avoid multiple obstacles in highly constrained environments and generated curvature-continuous collision-free trajectories based on a single-track vehicle model. The velocity profiles are applied by using convex optimization. However, these methods of constructing paths by polynomial curves often limit the shape of the trajectory and cannot find optimal solutions when driving in a complex environment with multiple obstacles.

After trajectory generation, the motion controller would combine the state of the actuator to produce a corresponding action. Currently, scholars mainly adopt a PID control strategy [23] for motion control, which has the advantage of independence from the accurate system model. However, how to reasonable design PID parameters and make them adaptive becomes difficult [24]. One variant of PID control, the pure tracking strategy [25], solved the problem of control parameter design, which was applied to the Navlab2V unmanned vehicle by Carnegie Mellon University [26]. On this basis, Kelly et al. [27] adjusted the pre-sighting distance according to the transverse error to make the trajectory smoother. However, pure tracking control cannot maintain the control performance under uncertainty of system parameters [28]. Fuzzy control [29] and model predictive control [30,31] are also widely used control strategies that do not rely on accurate system models. The former strategy design needs to be guided by extensive engineering experience, while the latter is able to consider both the vehicle model and the constraints of the control inputs [32]. Falone et al. [33,34] proposed a front wheel steering trajectory tracking control of autonomously driven vehicles based on MPC, calculating the desired steering wheel angle to track the trajectory in each step, and continuously improving the driving speed and testing its stability. Du X [35] proposed a nonlinear MPC controller using a genetic algorithm (GA) solver, using an Ackerman steering model and a predictive control algorithm, and using GA optimization to provide a more flexible structural formula for MPC. Katriniok A [36] used a simple linear two-degree-of-freedom model and a vehicle kinematics model to propose a model predictive control algorithm that considers soft constraint correction. The optimal control problem for each sampling time is converted into a quadratic programming problem, while the MPC controller can effectively reduce the tracking error and ensure the stability of the vehicle. Sun [37] proposed a human-like trajectory planning and tracking model based on a model predictive control algorithm, and which considers the drivers’ operational characteristics. Liu J [38] considered the tracking effect and steering stability simultaneously, based on a three-degree-of-freedom nonlinear vehicle model. However, a kinematic model with road coordinate can more accurately describe the relationship between the vehicle and reference road in unstructured environments.

In order to take safe actions for autonomous driving in unstructured environments, the main contributions of this paper consist of two aspects. First, by means of a nonlinear optimization method based on the kinematic model in the space domain, the original waypoints are smoothed into a reference road. To avoid irregular obstacles on unstructured roads, a multilayered search method in the lateral-space graph is introduced to find the safe path boundary in the current passable region. Then the lateral path is optimized by considering the lateral path boundary and the distance from the original reference road, as well as the curvature and the change of curvature. While, the optimized speed planner calculates the optimal speed profile by considering the speed boundary in the speed space domain and the constraints of acceleration. Second, an optimized motion controller based on the kinematic error model for road coordinates is proposed to give the lateral deviation and vehicle yaw error asymptotic stability, which can consider the constraints of the steering angle.

**Figure 1:**
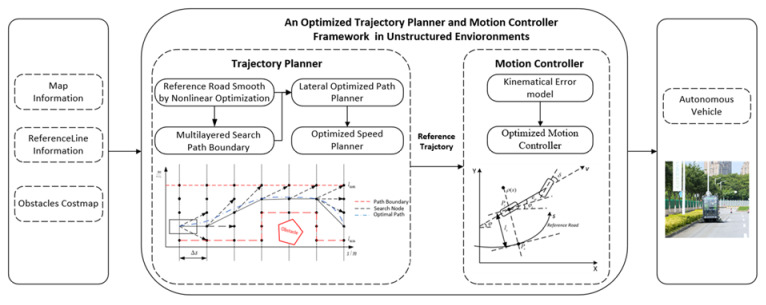
Trajectory planner and motion controller framework.

The remainder of the paper is organized as follows: The lateral path planner and the longitudinal speed planner in Section 2, the optimized motion controller in Section 3, experiment and discussions in Section 4, and conclusions and in Section 5. The trajectory planner and motion controller framework for unstructured environments of this paper are presented in Figure 1.

## 2. Optimized Motion Planner

The motion planner plays an important role in autonomous driving, connecting the perception system and motion control system to calculate the optimal driving behavior and desired trajectory. The system inputs include the reference path from the route module and the obstacle information from the perception module. There are some irregular obstacles in unstructured environments and these obstacles are described in an occupied cost map. The motion planner is decomposed of longitudinal planning and lateral planning sub-tasks to reduce the complexity of the problem. The lateral optimal trajectory is searched and optimized by using the kinematic model with the road coordinates and the optimal speed profile is calculated by considering the velocity limits and acceleration limits at each point.

### 2.1. Vehicle Kinematic Model with Road Coordinates

This section states the vehicle kinematic model with road coordinates system. First, the vehicle kinematic model in the space domain is introduced to describe the vehicle motion along the reference road. Then, the relative relationship between the vehicle and the reference road is established as a spatial-based model.

The shape of the plane curve is entirely determined by the curvature of each point, namely, s is the length of curve. Therefore, the plane curve with temporal properties can be replaced by the variable s to decouple the path from the velocity. The movement of a nonholonomic vehicle in the space domain can be represented as follows:

(1) $$\begin{array}{l} \frac{dx(s)}{ds}=\cos\varphi (s)\\ \frac{dy(s)}{ds}=\sin\varphi (s)\\ \frac{d\varphi (s)}{ds}=k(s) \end{array}$$

where *x(s)* and *y(s)* are the vehicle position, φ(s) is the vehicle heading, and *k(s)* is the vehicle curvature.

**Figure 2:**
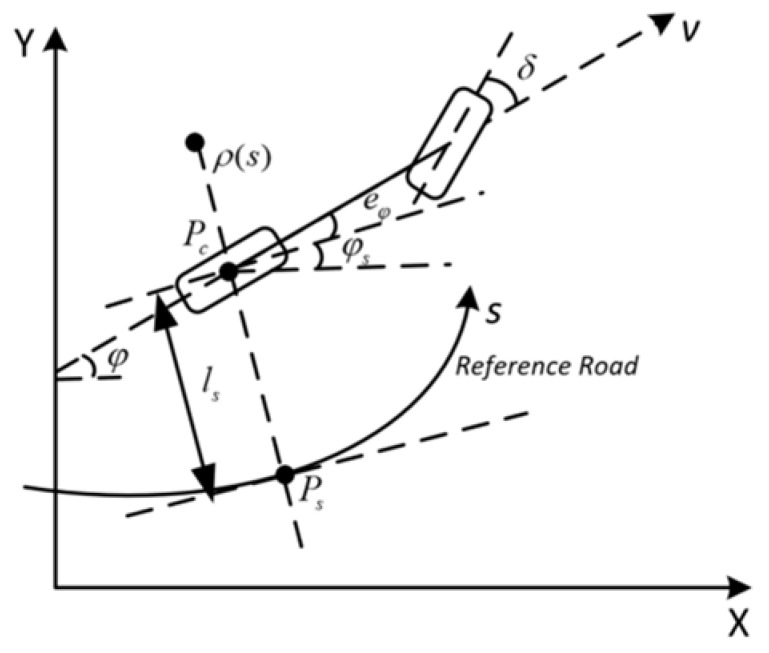
Vehicle kinematic model along the road coordinates.

The autonomous vehicles usually drive along the reference path. Unlike a vehicle kinematic model with global coordinates, we introduced the lateral error model with the road coordinates. The current point *P_c_* is located on the rear axle of the vehicle, and the tracked node *P_s_* is located in the given trajectory. The arc length *s* represents the distance along the reference path, and the lateral movement *l* and heading error $e_{\varphi }$ are modeled as a function of arc length *s* between the vehicle and the reference road, which are shown in Figure 2.

The relationship between the vehicle and road coordinate systems can be derived:

(2) $$\begin{array}{l} {\dot{l}}_{s}=v\sin(e_{\varphi })\\ {\dot{e}}_{\varphi }=\dot{\varphi }-{\dot{\varphi }}_{s}\\ \dot{s}=\frac{v\cos(e_{\varphi })\rho (s)}{\rho (s)-l_{s}} \end{array}$$

where ρ(s) is the radius of curvature of the reference road, φ and $\varphi_{s}$ are the vehicle yaw angle of the point *P_c_* and *P_s_* in the global coordinates. v is longitudinal speed of the vehicle.

In order to ignore the effect of longitudinal velocity on lateral path planning, Equation (2) can be derived as a function of arc length *s*.

(3) $$\begin{array}{l} l_{s}^{\prime }=\frac{{\dot{l}}_{s}}{\dot{s}}=\frac{\rho (s)-l_{s}}{\rho (s)}\tan(e_{\varphi })\\ e_{\varphi }^{\prime }=\frac{{\dot{e}}_{\varphi }}{\dot{s}}=\frac{(\rho (s)-l_{s})k}{\rho (s)\cos(e_{\varphi })}-\varphi_{s}^{\prime } \end{array}$$

where $l_{s}^{\prime }$ is the derivative of lateral offset with respect to arc length *s*, and $e_{\varphi }^{\prime }$ is the derivative of heading error with respect to arc length *s*.

The state variables of the vehicle model are $x={\left[\begin{array}{cc} l_{s} & e_{\varphi } \end{array}\right]}^{T}$, the control input is u=k, namely the curvature of the vehicle. To simplify the solution, the above nonlinear model is linearized and discretized by the reference point $k_{r}$. The equations can be obtained by solving the respective Jacobi matrices separately, the discretized system model is:

(4) $$x_{m+1}=A_{m}x_{m}+B_{m}u_{m}$$

(5) $$\left[\begin{array}{l} l_{s}(m+1)\\ e_{\varphi }(m+1) \end{array}\right]=\left[\begin{array}{cc} 1 & \Delta s\\ -k_{r}^{2}(m)\Delta s & 1 \end{array}\right]\left[\begin{array}{l} l_{s}(m)\\ e_{\varphi }(m) \end{array}\right]+\left[\begin{array}{c} 0\\ \Delta s \end{array}\right](k(m)-k_{r}(m))$$

where $x_{m+1}$ and $x_{m}$ are the discrete states at *m* and *m+1* step.

### 2.2. Reference Road Smoother

In unstructured environments, the reference path consists of a series of way points from the route module. Since the curvature of the reference path is usually discontinuous, the motion controller is difficult to track accurately. In order to consider the continuity of the first and second derivatives of the reference path, a nonlinear optimization algorithm based on the vehicle model in the space domain is employed to smooth the reference road.

The smoothing of the reference road is modelled as an optimization problem. The reference road, consisting of a series of discrete points, is given, and is defined as a vector ${\vec{X}}_{i}=(x_{i},y_{i})$. The number of discrete points is *N*. The objective function of the optimization problem has three components, which included the distance from the original reference point, the curvature of these discrete points, and the change of curvature. The objective function is defined as following:

(6) $$\begin{array}{l} f({\vec{X}}_{i})=\sum_{i=1}^{N}w_{ref}({\vec{X}}_{i}-{\vec{X}}_{ref}{)}^{2}+\sum_{i=2}^{N-1}w_{rk}(\frac{{\vec{X}}_{i-1}-2{\vec{X}}_{i}+{\vec{X}}_{i+1}}{\Delta s^{2}}{)}^{2}\\ \begin{array}{ccc} & & \sum_{i=2}^{N-2}w_{rj}(\frac{-{\vec{X}}_{i-1}+3{\vec{X}}_{i}-3{\vec{X}}_{i+1}+{\vec{X}}_{i+2}}{\Delta s^{3}}{)}^{2} \end{array} \end{array}$$

where ${\vec{X}}_{ref}$ is the original reference point, $w_{ref}$ is the weighting factor for distance from the original reference point, and $w_{rk}$ and $w_{rj}$ are the weighting factors of curvature and curvature change, respectively.

At the same time, the variation of two adjacent points on the reference road needs to satisfy the constraints of the vehicle model. We used the vehicle model in the space domain. Then the discrete vehicle model is as follows:

(7) $$\begin{array}{l} x_{i+1}(s)=x_{i}(s)+\Delta s\cos\varphi_{i}(s)\\ y_{i+1}(s)=y_{i}(s)+\Delta s\sin\varphi_{i}(s)\\ \varphi_{i+1}(s)=\varphi_{i}(s)+\Delta sk_{i}(s) \end{array}$$

Moreover, these discrete points must not exceed the road boundary. The constraints on the road boundary are as follows:

(8) $$\left[\begin{array}{c} x_{i}^{\min}\\ y_{i}^{\min}\\ \varphi_{i}^{\min} \end{array}\right]<<\left[\begin{array}{c} x_{i}\\ y_{i}\\ \varphi_{i} \end{array}\right]<<\left[\begin{array}{c} x_{i}^{\max}\\ y_{i}^{\max}\\ \varphi_{i}^{\max} \end{array}\right],i=1,2,3\ldots \ldots N$$

where $\left(x_{i}^{\min},y_{i}^{\min},\varphi_{i}^{\min}\right)$ and $\left(x_{i}^{\max},y_{i}^{\max},\varphi_{i}^{\max}\right)$ are the left and right road boundary, respectively.

The curvature of the road is limited as:

(9) $$k_{\min}<<k_{i}<<k_{\max},i=1,2,3\ldots \ldots N$$

Therefore, according to the above objective function and constraints, this nonlinear optimization problem can be implemented by using Interior Point Optimize (Ipopt) solver [19], which is a software package for large-scale nonlinear optimization.

**Figure 3:**
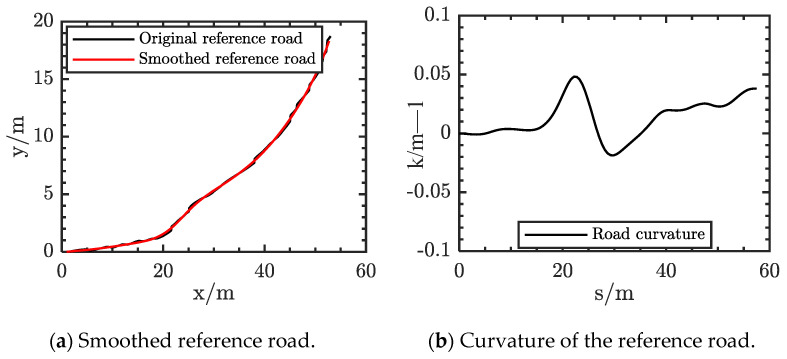
Results of reference road smoothing.

The result of a smoothed example is shown in the Figure 3a. The black line is the original reference road, which is very uneven and has a lot of burrs in the graph. The smoothed reference road is shown as a red line. In addition, the curvature of the reference road is shown in the Figure 3b. Its smoothness meets the requirements of the vehicle.

### 2.3. Multilayered Search Path Boundary

In order to ensure driving safety in unstructured environments, it is necessary to generate an optimal trajectory along the road geometry. The vehicle motion is decomposed into longitudinal and lateral movements along the reference path. The lateral optimized path planner is introduced to avoid different irregular obstacles in the unstructured environment. Thus, the boundary of the path needs to be determined before the optimization. To achieve this, the road geometry is described in road coordinates rather than the Cartesian coordinate framework. The smoothed reference path is represented as the base frame. Based on the road coordinates, there are two parameters of (*s*,*l*). Where *s* is the longitudinal distance along the reference path, and the lateral deviation *l* is the vertical distance between the current position and the nearest point in the reference path.

**Figure 4:**
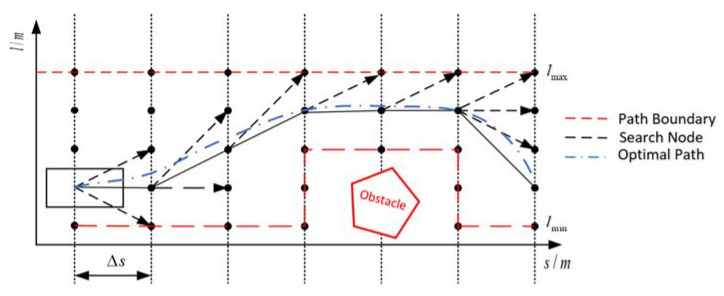
Multilayered Search Path Boundary in Lateral-Space graph.

The multilayered search method is applied to find a suitable path boundary. The traditional search method is difficult for considering the lateral movement constraints of the vehicle, it is usually searched in Cartesian coordinates. The idea of this search algorithm is derived from a dynamic programming algorithm. In this work, we used a lateral motion model to search the road coordinate system. It needs to be sampled at equal distance intervals along the reference path to form multiple layers. The static obstacles are shown as grey regions in Figure 4.

In order to avoid static obstacles in the grey region, the multilayered search algorithm is introduced. First, we calculated the current vehicle position in the base frame. The reference path is divided into multiple layers and the longitudinal spacing of the grid is Δs. From the first point (*s*_0_,*l_0_)*, the expansion of the node is used by the lateral movement model. Moreover, the candidate points must satisfy the lateral constraints. The candidate nodes that do not meet the constraint are removed from the set. This can be represented as Equations (10) and (11).

(10) $$l_{\min}^{\prime }\leq \frac{l_{i}-l_{i-1}}{\Delta s}\leq l_{\max}^{\prime }$$

(11) $$l_{\min}\leq l_{i}\leq l_{\max}$$

where $l_{\min}^{\prime }$ and $l_{\max}^{\prime }$ are the minimum and maximum of the derivative of lateral distance. $l_{\min}$ and $l_{\max}$ are the left and right road boundary in the unstructured environments.

Second, each candidate point has a priority cost and heuristic cost. The priority cost is comprised of five weighted cost terms. The cost is calculated by the weight of the sum of the reference cost *g_r_*, the obstacle cost *g_o_*, the consistency cost *g_c_*, the lateral acceleration cost *g_a_*, and the jerk cost *g_j_*. This is written as in Equation (12). The heuristic cost is the longitudinal distance with the goal.

(12) $$g_{l}=w_{r}g_{r}+w_{o}g_{o}+w_{c}g_{c}+w_{a}g_{a}+w_{j}g_{j}$$

where $w_{r}$, $w_{o}$, $w_{c}$, $w_{a}$, and $w_{j}$ are the weight coefficients, respectively.

To ensure that the vehicle does not move away from the reference road, the reference cost is defined as Equation (13), and $l_{ref}$ is the reference lateral distance.

(13) $$g_{r}={\left(l_{i}-l_{ref}\right)}^{2}$$

The penalty cost of the obstacle ensures that the path can maintain a safe distance from the obstacles during the process of searching, which is designed as follows:

(14) $$g_{o}=\left\{ \begin{array}{l} {\left(d_{\max}-d_{obs}\right)}^{2},d_{obs}\leq d_{\max}\\ \begin{array}{cc} 0, & \begin{array}{cc} & otherwise \end{array} \end{array} \end{array}\right.$$

where $d_{obs}$ is the distance from the node to the nearest obstacle and $d_{\max}$ is the safe distance.

At the same time, the consistency cost is designed to ensure the continuity of planning. It can be represented as follows:

(15) $$g_{c}={\left(\frac{l_{i}-l_{i-1}}{\Delta s}\right)}^{2}$$

The penalties on lateral acceleration and jerk related to the driving comfort are expressed as follows:

(16) $$g_{a}={\left(\frac{l_{i}{}^{\prime }-l^{\prime }{}_{i-1}}{\Delta s}\right)}^{2}$$

(17) $$g_{j}={\left(\frac{l^{\prime }{}_{i}+l_{i-2}^{\prime }-2l_{i-1}{}^{\prime }}{\Delta s^{2}}\right)}^{2}$$

Third, we initialized an open set and close set and added the start node into the open set. During the search performed at each layer, there are a list of nodes, it is necessary to check the collision with the static obstacles in the cost map, and if a collision occurs they cannot be added to the open set. Furthermore, the cost of each node consists of a priority cost and heuristic cost. The heuristic cost is defined as the longitudinal distance with the goal, namely $h_{i}={\left(s_{i}-s_{target}\right)}^{2}$. According to the cost of each layer, we selected the node with the smallest cost from the open set as the new node for the next expansion step. Based on the graph search method, it will keep repeating the above steps until the target longitudinal distance is reached. Then, the searched node is traced step by step from the end node until the starting node is reached.

Finally, based on the final result of the search, we calculated the distance from the obstacle for each point on each layer and selected the safety boundary point. Then, the path boundary was found along the reference path.

### 2.4. Optimized Path Planner

In this section, a lateral optimized path planner is introduced to calculate the optimal trajectory. The path planning problem is essentially a multi-objective optimization problem. It needs to consider the distance to the obstacle, the distance to the reference line, and the constraints of the vehicle model. The lateral path optimization makes it easier to consider the shape of the road. The length of the planned path increases with the speed of the vehicle and is divided equally into *N* steps, namely s=NΔs. The lateral constraints are obtained from the previous path boundary to avoid obstacles along the reference path. Thus, the path optimization problem is established as follows:

(18) $$\begin{array}{l} \min J_{l}=\sum_{i=0}^{N}w_{l}(l_{i}-l_{ref}{)}^{2}+\sum_{i=0}^{N}w_{\theta }(l_{i}^{\prime }-l_{ref}^{\prime })+\sum_{i=0}^{N-1}w_{k}u_{i}^{2}\\ x_{i+1}=A_{i}x_{i}+B_{k}u_{i},\begin{array}{cc} & i=0,1,2\ldots \ldots N-1 \end{array}\\ l_{\min}\leq l_{i}\leq l_{\max},\begin{array}{cc} & i=0,1,2\ldots \ldots N \end{array}\\ u_{\min}\leq u_{i}\leq u_{\max},\begin{array}{cc} & i=0,1,2\ldots \ldots N \end{array} \end{array}$$

where $l_{\min}$ is the lateral minimum boundary and $l_{\max}$ is the lateral maximum boundary. While, the control input is the curvature of path, namely $u_{k}=k_{k}$. $u_{\min}$ and $u_{\max}$ are the minimum curvature and maximum curvature limits. $w_{l}$, $w_{\theta }$, and $w_{k}$ are the weighting coefficients of the lateral distance with the reference path, the relative angle with the reference path, and control inputs, respectively.

In this work, the objective function and the constraint functions were converted into the quadratic form. The lateral optimal problem can be implemented by using the operator splitting quadratic program (OSQP) solver with low computation.

Finally, the optimal points $\left(s_{i},l_{i}\right)$ are obtained along the base frame. According to the longitudinal distance *s_i_*, it is necessary to find the corresponding reference point $\left(x_{i}^{r},y_{i}^{r},\varphi_{i}^{r}\right)$. Furthermore, these points are converted by using Equation (10) in the Cartesian coordinate system to calculate the desired path.

(19) $$\begin{array}{l} x_{i}(s_{i},l_{i})=x_{i}^{r}+l_{i}\cos(\varphi_{i}^{r}+\frac{\pi }{2})\\ y_{i}(s_{i},l_{i})=y_{i}^{r}+l_{i}\sin(\varphi_{i}^{r}+\frac{\pi }{2})\\ \varphi_{i}(s_{i},l_{i})=\varphi_{i}^{r}-\varphi_{i}^{s}\\ \varphi_{i}^{s}=dl_{i}/ds_{i} \end{array}$$

where $x_{i}(s_{i},l_{i})$, $y_{i}(s_{i},l_{i})$, and $\varphi_{i}(s_{i},l_{i})$ are the optimal points in Cartesian coordinates. $\varphi_{i}^{s}$ is the first-order derivative of lateral motion with longitudinal distance.

### 2.5. Optimized Speed Planner

**Figure 5:**
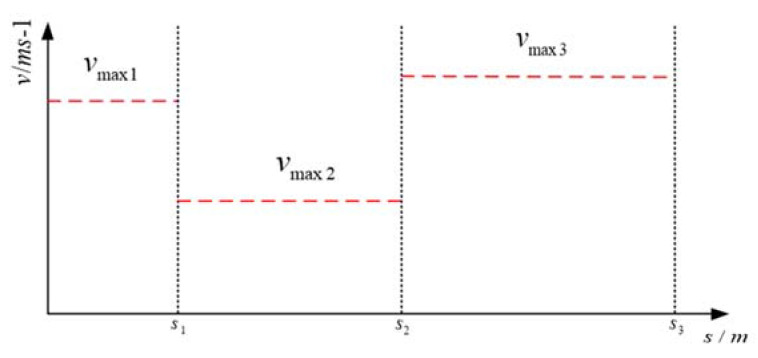
Speed Limit along the Desired Path.

After calculating the desired path, speed planning is performed at each point. The optimized speed planner is applied to achieve the desired speed in the unstructured environment. Each point on the reference path has a different speed limit, such as deceleration being required where the curvature is too large to meet the lateral acceleration constraint. At the same time, the vehicle motion needs to satisfy the acceleration and deceleration constraints. The speed limits at each longitudinal distance are shown in Figure 5, where $v_{\max1}$, $v_{\max2}$, and $v_{\max3}$ are the speed limits at different stages.

The maximum velocity depends on the desired velocity from the decision module and the maximum curvature of the reference point.

(20) $$v_{\max k}\leq a_{y}/k_{\max}$$

(21) $$v_{\max}=\min(v_{\max}^{k},v_{\max}^{ref})$$

The longitudinal motion description can be described as in Equation (22), we can represent *v* as a function of *s*.

(22) $$v_{i}^{2}=v_{i-1}^{2}+2a_{i}\Delta s$$

where $a_{i}$ is the longitudinal acceleration of the vehicle.

Similarly to the lateral path optimized method, the details of the optimized speed planner will be introduced in the following sections. The speed planner has the responsibility for generating a speed profile considering the target speed, safety constraints, and vehicle acceleration limits. Therefore, we generated an optimal speed profile in the space-domain to consider the speed limit more precisely. The entire path is uniformly segmented into *N* steps, and the length of each step is Δs. The speed optimized problem can be expressed as Equation (23). To simplify the problem, we defined $M_{i}=v_{i}^{2}$.

(23) $$\begin{array}{l} \min J_{s}=\sum_{i=0}^{N}(M_{i}-M_{ref}{)}^{2}+\sum_{i=0}^{N-1}a_{i}^{2}\\ \begin{array}{ccc} & M_{i}=M_{i-1}+2a_{i}\Delta s & i=1,2,3\ldots \ldots N \end{array}\\ \begin{array}{cc} & \begin{array}{cc} v_{\min}^{2}\leq M_{i}\leq v_{\max}^{2}, & i=1,2,3\ldots \ldots N \end{array} \end{array}\\ \begin{array}{ccc} & a_{\min}\leq a_{i}\leq a_{\max}, & i=1,2,3\ldots \ldots N-1 \end{array} \end{array}$$

Similarly, this optimization problem is also converted to the quadratic form and uses the OSQP solver to calculate the optimal speed profile.

Finally, we interpolated by distance to get the velocity of each point. Furthermore, the optimal trajectory is obtained and sent to the motion controller module.

## 3. Optimized Motion Controller

In this section, an optimized motion controller based on the vehicle kinematic model for road coordinates was established to track the reference trajectory from the planning module. The proposed motion controller can provide the optimal control command. First, the kinematic error model with the road coordinates is linearized and discrete to simplify the solution. Then, the optimization problem of control is designed by considering the lateral deviation and heading deviation for the desired path. Finally, the optimized motion controller performs the lateral deviations and heading error asymptotic stability adjustments and meets the constraints of the steering angle at the same time.

The kinematic error model for road coordinates can more accurately describe the relationship between the vehicle and reference road. It can be represented with Equation (2), as follows:

(24) $$\begin{array}{l} e_{y}=v\sin(e_{\theta })\\ e_{\theta }=v\tan\sigma /l-vk_{r} \end{array}$$

where $e_{y}$ and $e_{\theta }$ are the lateral error and heading deviation in the desired trajectory frame. δ is the steering angle and $k_{r}$ is the reference curvature at the desired trajectory.

For the formulation of a linear quadratic programming optimization, we linearized the vehicle model by using first-order Taylor approximation around the reference inputs. This can be inferred as follows:

(25) $$\left[\begin{array}{l} e_{y}(k+1)\\ e_{\theta }(k+1) \end{array}\right]=\left[\begin{array}{cc} 1 & v_{r}\\ 0 & 1 \end{array}\right]\left[\begin{array}{l} e_{y}(k)\\ e_{\theta }(k) \end{array}\right]+\left[\begin{array}{c} 0\\ v_{r}/l{\left(\cos\delta_{r}\right)}^{2} \end{array}\right](\delta -\delta_{r})+\left[\begin{array}{c} 0\\ -v_{r}k_{r} \end{array}\right]$$

where $\delta_{r}$ is the reference steer angle.

The model predictive control method can simultaneously consider the mathematical model of the controlled object system and establish safe constraints to calculate optimal control inputs. In order to take advantage of the desired trajectory in the future, we used a kinematic error model to predict the deviation in the future. At the same time, the constraint on the actuator is introduced. The optimal motion control problem based on the model predictive control subject to track the reference trajectory and steering limitations is designed as follows:

(26) $$\begin{array}{l} J(y(k),u(k))=\sum_{i=0}^{N_{p}}{\left\|Q(y(k+i|k)-y_{ref}(k+i|k))\right\|}^{2}\\ +\sum_{i=0}^{N_{p}-1}{\left\|Ru(k+i|k)\right\|}^{2}\\ \begin{array}{ccc} & u_{\min}\leq u_{i}\leq u_{\max}, & i=1,2,3\ldots \ldots N_{p}-1 \end{array} \end{array}$$

where *Np* is the prediction steps and the system output is $y(k)={\left[\begin{array}{cc} e_{y} & e_{\theta } \end{array}\right]}^{T}$. $Q=\left[\begin{array}{cc} Q_{l} & 0\\ 0 & Q_{\theta } \end{array}\right]$ is the parameter of the optimization motion controller, and $Q_{l}$ and $Q_{\theta }$ are the weight coefficient of lateral error and heading deviation, respectively. *R* is the weight coefficient of steering angle.

The transformation of the above formula can use the quadratic programming function to optimize the objective function to obtain the current optimal steering wheel angle.

## 4. Experiments and Discussion

**Figure 6:**
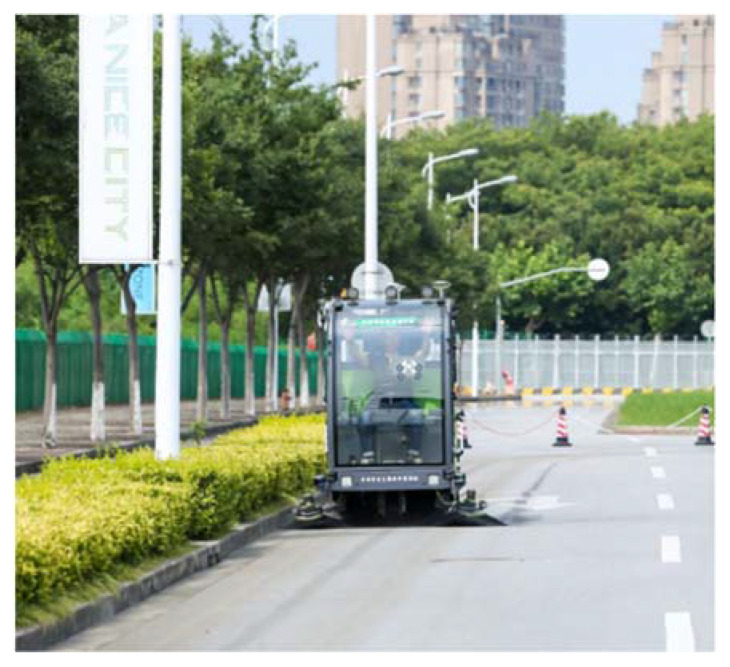
Autonomous sweeper.

To test the validity of the presented method in unstructured environments, we applied it to a self-driving sweeper. As shown in Figure 6, the autonomous sweeper is designed to collect rubbish, such as leaves, dust, and so on, along the road curb. The vehicle is equipped with a high-accuracy positioning system, visual and lidar perception system, drive-by-wire chassis, decision-planning-controlling module, and remote monitoring and scheduling system. Experiments were conducted in the Ubuntu 18.04 LTS environment with C++ language and executed on an Intel Core i7-4200 H@2.8 GHz with 8.00 GB RAM.

And key parameters are listed in Table 1.

The environment around the sweeper was expressed by an occupied grid map with a grid size of 400 × 400 and resolution of 0.1 m/grid. In order to verify the real-time performance and safety of the algorithm, three typical scenarios of obstacle avoidance were designed. Moreover, the results of the proposed method were compared with the traditional hybrid A star algorithm [6].

**Figure 7:**
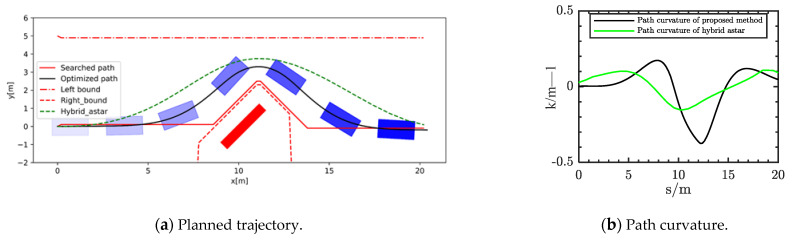
Planned result in scenario 1.

Scenario one was single obstacle experiment with a rectangle obstacle on the reference road. The size of the obstacle was 3.0 × 0.5 m. As shown in Figure 7a, the planned path of our method could avoid the obstacles without collisions. At the same time, it was not far from the reference road. The safe driving bounds were searched through multilayer sample points. The right bound could accurately envelop the obstacle within the safety distance. Meanwhile, the planning result of hybrid A star is shown by the green line in the Figure 7. It can be seen that these results are further away from the reference line. Moreover, the curvature of the planned path is shown in the Figure 7b. It can be seen that the optimized path can meet the requirements of comfort of the vehicle.

**Figure 8:**
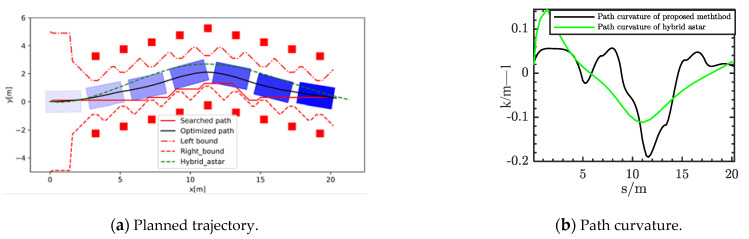
Planned result in Scenario 2.

In order to verify the avoidance ability of the proposed method in a narrow space with dense obstacles, the simulation scenario two was designed as in Figure 8. There are many different obstacles placed around the reference road, which are shown as red rectangles. The size of the obstacles was 0.5 m × 0.5 m. This is a typical scene in unstructured environments. The multilayer searched path could avoid the obstacles and determine the direction of obstacle avoidance. These optimal nodes can satisfy the designed objective function. These boundary lines became jagged because the safety distance between the two obstacles was larger. We calculated the safety distance with the obstacle to determine whether it could be passed. Furthermore, the safe driving corridor could maintain a certain safe distance from all the obstacles in current passable region. The results in Figure 8 show that the points of the optimal solution were within the upper and lower boundaries. At the same time, the curvature of the optimized path is shown in the Figure 8b, satisfying the kinematic constraints of the vehicle. The maximum curvature during the whole obstacle avoidance process reached close to 0.2. The curvature varied more smoothly than hybrid A star. As a result, the autonomous vehicle could avoid all the obstacles and then return to the target reference road.

**Figure 9:**
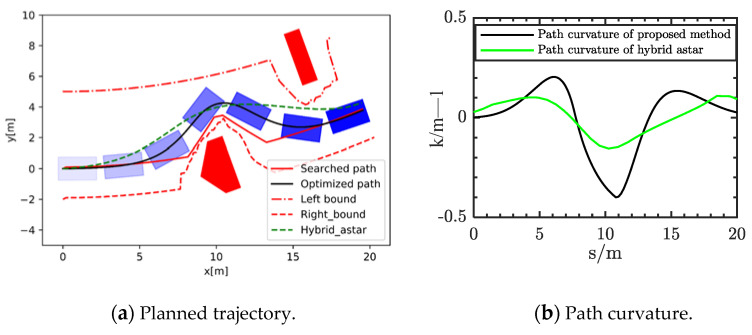
Planned result in Scenario 3.

An environment with a curvy road was designed as in Figure 9. There are two obstacles on both sides of the reference road and each obstacle takes up half the width of the lane. The ego vehicle can also avoid two separate obstacles while satisfying the vehicle curvature constraint of the curvy road. We created safe driving corridors along the direction of the road through the searched path. The optimized result is shown as the black line in Figure 9a. The change of curvature during the whole process is shown in Figure 9b. The maximum curvature of the path reaches 0.4. Although the curvature is larger than the result of hybrid A star, it can ensure the safety of the trajectory within the certain safe distance. In addition, the discrete sampling distance of the reference line was 0.2 m. As the sampling distance decreases, the optimized trajectory will be smoother, but the planning operation time will increase accordingly. The ego vehicle avoids obstacles and returns to the reference road as soon as possible.

**Figure 10:**
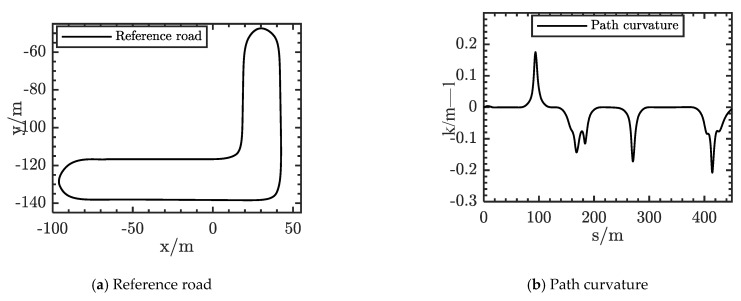

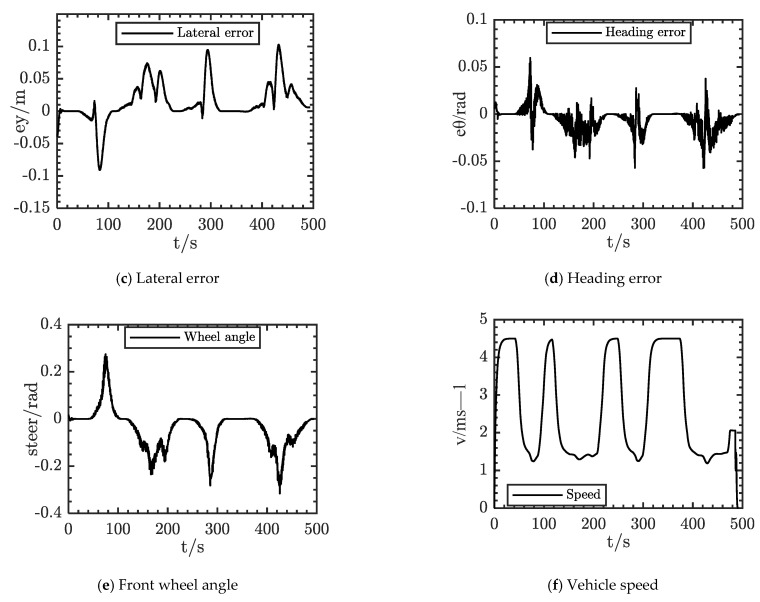
Vehicle control input.

Through these three simulation cases, the proposed method in this paper was verified, and could find the optimal path to avoid collisions in unstructured environments. The maximum iteration size of the optimization solution was limited to 1000 times. The average computation time was less than 50 ms. The proposed lateral path optimization method in this paper could meet the real-time requirements. As a result, the proposed lateral optimization planner can generate both complex maneuvers and smooth paths. In order to verify the validity of the motion controller, some typical experiments were designed. These were straight lines, left turn, right turn, and U-turn in this scenario. The reference road of this scenario is shown in Figure 10a,b, where the maximum curvature was nearly 0.2. The length of the reference road was nearly 425 m.

The lateral displacement error and heading angle error are shown in Figure 10c,d. From the graph, it can be seen that the optimized motion controller could effectively guarantee the convergence of the heading angle deviation and the lateral displacement deviation. The average lateral deviation during the whole process was less than 15 cm. The maximum lateral deviation was 12 cm at the U-turn, which was due to the larger curvature. The maximum heading angle deviation did not exceed 5 degrees. It could meet the requirements of the motion planner. The wheel angle and the vehicle speed are shown in Figure 10e,f. The maximum front wheel rotation angle reached 18 degree at the U-turn. It could meet the maximum steering angle limit. The speed of the reference point was 4.5 m/s. However, due to the limitation of lateral acceleration, the speed was limited to 1.5 m/s during the turn. The prosed method can slow down in advance before turning. At the same time, it can satisfy the constraints of the control input.

As a result, the tracking effect of the proposed motion controller was proven. The predicted step size was 30 and the average computation time was less than 30 ms during the whole process. This can meet the real-time requirement and guarantee the stability of the vehicle.

## 5. Conclusions

An optimized trajectory planner and motion controller framework were proposed in order to ensure the safety of autonomous driving in unstructured environments. On the one hand, the nonlinear optimization method was applied to smooth the reference road based on the vehicle kinematic model in the space domain. The trajectory planning consisted of longitudinal and lateral planning sub-tasks. The multilayered search method in the lateral-space graph created safe driving corridors to avoid obstacles. Furthermore, the lateral optimized path was obtained by considering the distance to the reference road and the smoothness of the path. Similarly, the optimal speed profile was optimized using a speed-space graph to achieve the desired velocity and simultaneously meet the acceleration constraints. On the other hand, an optimized motion controller based on a vehicle kinematic model was introduced to track the desired trajectory. The results illustrate that the proposed trajectory planner and motion controller framework can handle different situations and keep safe in unstructured environments. It can smoothly and safely avoid different irregular obstacles. In addition, the optimized motion controller has less overshoot and lateral errors.

Future work will focus on considering the uncertainty of perception during the process of motion planning. Furthermore, we will apply the proposed method to more dynamic road traffic scenarios.

## Figures and Tables

**Table 1:** Key parameters.

Parameter	Value	Parameter	Value	Parameter	Value	Parameter	Value
Sweeper Length	2.22 m	$w_{ref}$	0.4	$w_{o}$	0.1	$Q_{l}$	500
Sweeper Width	1.60 m	$w_{rk}$	0.3	$w_{c}$	0.2	$Q_{\theta }$	100
*l*	1.34 m	$w_{rj}$	0.3	$w_{a}$	0.2	R	1000
$\delta_{\max}$	40°	$w_{r}$	0.2	$w_{j}$	0.3	$N_{p}$	30

## Data Availability

Not applicable.
